# Antibacterial Effect of Copper on Microorganisms Isolated from Bovine Mastitis

**DOI:** 10.3389/fmicb.2016.00626

**Published:** 2016-04-28

**Authors:** Angelica Reyes-Jara, Ninoska Cordero, Juan Aguirre, Miriam Troncoso, Guillermo Figueroa

**Affiliations:** ^1^Laboratorio de Microbiología y Probióticos, Instituto de Nutricion y Tecnologia de los Alimentos, Universidad de ChileSantiago, Chile; ^2^Departamento de Nutricion, Bromatología y Tecnología de los Alimentos, Facultad de Veterinaria, Universidad Complutense of MadridMadrid, Spain

**Keywords:** copper, antimicrobial activity, milk, bovine mastitis

## Abstract

The antimicrobial properties of copper have been recognized for several years; applying these properties to the prevention of diseases such as bovine mastitis is a new area of research. The aim of the present study was to evaluate *in vitro* the antimicrobial activity of copper on bacteria isolated from subclinical and clinical mastitis milk samples from two regions in Chile. A total of 327 microorganisms were recovered between March and September 2013, with different prevalence by sample origin (25 and 75% from the central and southern regions of Chile, respectively). In the central region, *Escherichia coli* and coagulase negative *Staphylococci* (CNS) were the most frequently detected in clinical mastitis cases (33%), while in the southern region *S. uberis*, *S. aureus*, and CNS were detected with frequencies of 22, 21, and 18%, respectively. Antibiotic susceptibility studies revealed that 34% of isolates were resistant to one or more antibiotics and the resistance profile was different between bacterial species and origins of isolation of the bacteria. The minimum inhibitory concentration of copper (MIC-Cu) was evaluated in all the isolates; results revealed that a concentration as low as 250 ppm copper was able to inhibit the great majority of microorganisms analyzed (65% of isolates). The remaining isolates showed a MIC-Cu between 375 and 700 ppm copper, and no growth was observed at 1000 ppm. A linear relationship was found between the logarithm of viable bacteria number and time of contact with copper. With the application of the same concentration of copper (250 ppm), CNS showed the highest tolerance to copper, followed by *S. uberis* and *S. aureus*; the least resistant was *E. coli*. Based on these *in vitro* results, copper preparations could represent a good alternative to dipping solutions, aimed at preventing the presence and multiplication of potentially pathogenic microorganisms involved in bovine mastitis disease.

## Introduction

Bovine mastitis is an inflammatory disease of the mammary gland (Bradley, 2002; Thompson-Crispi et al., 2014); it represents one of the main production and economic problems confronting the global dairy industry (Zadoks and Fitzpatrick, 2009). The high costs associated with this frequent pathology are derived from its impact on animal health and welfare and the profitability of milk sales (i.e., impacts on milk production level and milk quality) (O’Grady and Doherty, 2009). International studies estimate an average cost of U.S. $224-275 per case, resulting in annual losses in the millions, including costs of antibiotic treatments, veterinary care, milk withdrawal, labor, and milk production losses (Steeneveld et al., 2011).

Bovine mastitis disease begins with the invasion and colonization of microorganisms via the teat duct orifice, creating inflammation in the mammary gland that results in a clear productive decline and in unwanted physical and chemical changes in the milk (De Vliegher et al., 2012; Thompson-Crispi et al., 2014). *Staphylococcus aureus*, *Escherichia coli*, and *Streptococcus uberis* are frequently found in clinical mastitis (De Vliegher et al., 2012). Intramammary bacterial infections are the principal causes of clinical and subclinical mastitis (De Vliegher et al., 2012).

The clinical manifestation is characterized by physical alterations of the gland; this in turn deteriorates milk quality and reduces its volume (Cha et al., 2011). In contrast, in subclinical mastitis the visual symptoms and signs are absent, but the milk shows elevated somatic cell counts (SCCs) (Halasa et al., 2009; De Vliegher et al., 2012), which reduces the value of the milk. In addition, subclinical mastitis may also cause infection by spreading the bacteria among farms and even between animal species.

The National Institute for Research in Dairying (NIRD) program includes different strategies for mastitis control management such as dry cow treatment, milking techniques, teat disinfection with topical antiseptic substances (pre-dipping and dipping) and antibiotic treatment of clinical mastitis cases (Barkema et al., 1998; Nickerson, 2009). However, antiseptic substances routinely used in dairy production such as iodine or chlorhexidine are not completely effective in reducing or preventing mastitis (Drechsler et al., 1993; El Behiry et al., 2012).

The use of antibiotics, confined to selected severe cases, requires bacterial isolation and antibiotic selection (Roberson, 2012). In addition, the routine use of antibiotics is questionable because their use can generate unwanted residues not accepted in milk, and because they can spread the emergence of antimicrobial-resistant strains (Gao et al., 2012; Oliver and Murinda, 2012; Wang et al., 2012; Nosanchuk et al., 2014). Since bovine mastitis remains a worldwide problem, producers and governments continue searching for a non-antibiotic solution or technologies to reduce the prevalence of the disease (Cheng et al., 2014).

Finding successful strategies for the control of bovine mastitis is a challenge for dairy producers. Currently the programs are based on hygiene and include teat disinfection, antibiotic therapy and culling of chronically infected cows. The search for alternatives to antibiotics (i.e., bacteriophages, vaccines, or natural compounds) is a field being explored to find an effective approach for management of bovine mastitis (Gomes and Henriques, 2016). One alternative to be used as an effective teat disinfection may be a copper-based product.

The use of antimicrobial copper was accepted for the first time in 2008 by the United States Environmental Protection Agency (EPA, 2008)^1^. Studies showed that copper surfaces can eliminate bacteria (i.e., *S. aureus, Enterobacter aerogenes*, MRSA*, Pseudomonas aeruginosa*, and *E. coli* O157:H7) usually causing nosocomial infections. Lately, the efficacy of copper has been tested in other microorganisms such as viruses, fungi, and other bacterial pathogens (Faúndez et al., 2004; Wilks et al., 2005; Noyce et al., 2006; Grass et al., 2011).

Copper antibacterial functionality is associated with various mechanisms, including damaging the microbial DNA, altering bacterial protein synthesis and altering membrane integrity (Warnes et al., 2010; Grass et al., 2011; Chaturvedi and Henderson, 2014). In addition, the antibacterial effect of copper was already proved for *E. coli* and *S. aureus*, two of the main bacterial species involved in mastitis (Noyce et al., 2006; Espírito Santo et al., 2011).

The aim of the present study was to evaluate *in vitro* the antimicrobial activity of copper on microorganisms isolated from bovine mastitis. Microorganisms were isolated from milk samples of animals with bovine mastitis (clinical and subclinical). Samples were from two geographic areas of Chile (central and southern regions) with different production systems between March and September, 2013.

## Materials and Methods

### Sampling and Bacterial Identification

**FIGURE 1:**
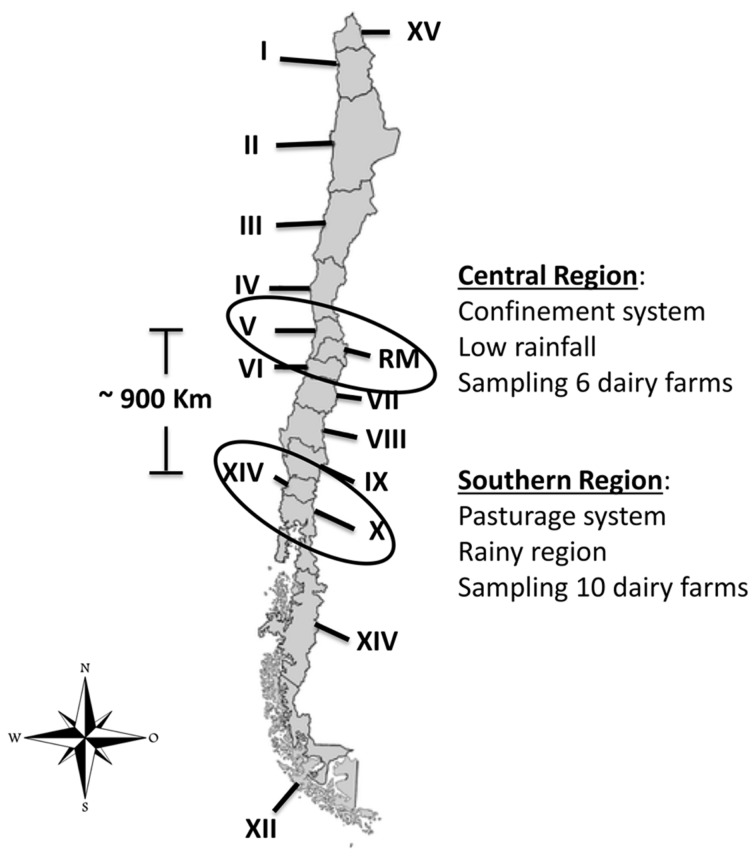
**Location in Chile of dairy farms sampled (López, 2014).** Chile is divided into 15 Regions, each one is identified by a Roman numeral (I to XV). Regions sampled during the study are enclosed in circles.

We analyzed 386 samples of raw milk from clinical and subclinical bovine mastitis that were collected between March and September 2013 in two geographical regions of Chile (central and southern). A total of sixteen dairy farms were included in the sampling, six from the central region and ten from the southern region (**Figure 1**). The selected cows were previously diagnosis with clinical or subclinical bovine mastitis and the sampling conducted during regular milking times.

Ethical approval was not required for milk sampling, because this study was carried out in accordance with the routinely milking procedures, which was supervised by official veterinarians. Subclinical and clinical bovine mastitis were defined according to Schukken et al. (2003). In subclinical mastitis, the SCC was greater than 200,000 cells per milliliter of milk. In the case of clinical mastitis, in addition to high SCC the milk presented evident organoleptic changes, such as the presence of clots and yellow color, among others, which were visually inspected.

Tested animals were under a regular disinfection protocol using iodine as teat dip before milking. Milk samples from each teat were collected aseptically according to the NMC protocol, placed into sterile tubes and transported on the same day under cooling conditions to the laboratory. Bacteriological culture of milk samples and bacterial identification were performed as recommended by the National Mastitis Council.

The collected milk samples were centrifuged at 2500 × *g* for 5 min and the pellet was cultured on different agar media: 5% sheep blood agar plates, McConkey agar plates (Oxoid Ltd, Basingstoke, Hampshire, England), tryptic soy agar plates (BBL, Becton Dickinson, USA) and Sabouraud dextrose agar. Ten micro liter of pellet were streaked per agar plate. The agar plates were incubated at 37°C for 18–24 h; those with no growth were left up to 48 h. The results obtained in the culture medium were interpreted and recorded according to the following criteria: (a) positive culture: growth of five or more colonies of the same type or two types of colony with a minimum eight colonies per type. (b) Negative culture: no growth or growth of less than five colonies of the same type. (c) Contaminated sample: growth of three or more different types of colonies. Isolates were sub-cultured to obtain pure cultures. The identification of bacteria was performed according to colony morphology, Gram staining, and biochemical tests.

Phenotypic differentiation of bacterial species was conducted as follows: *Staphylococci* and *Streptococci* were identified presumptively based on colony morphology, Gram stain and catalase test. Coagulase-positive *Staphylococci* (*S. aureus*, *Staphylococcus intermedius*, and *Staphylococcus hyicus*) were identified based on morphology, pigmentation, coagulase test, Voges Proskauer, and the ONPG test (Capurro et al., 1999). Specific polymerase chain reaction (PCR) tests were used to confirm *S. aureus* strains using primers NUC1 and NUC2 described by (Gandra et al., 2011) (**Supplementary Figure S1A**).

All coagulase-negative *Staphylococci* were considered as CNS (coagulase-negative *Staphylococci*) after showing resistance to bacitracin (0.04U). The *Staphylococci* sensitive to bacitracin (0.04U) were considered as *Micrococcus* spp. (Falk and Guering, 1983). *Streptococcus* species were differentiated based on hemolysis, esculin, CAMP and the inulin test. *S. uberis* was confirmed by PCR with oligonucleotide primers designed for the 16S rRNA gene (Hassan et al., 2001) (**Supplementary Figure S1B**).

*Corynebacterium* spp. were identified presumptively based on colony morphology, hemolysis, esculin, and the API^®^ Coryne test (Biomerieux). Results were confirmed by PCR amplification of the *rpoB* gene (Khamis et al., 2004) (**Supplementary Figure S1C**). Yeasts and Enterobacteriaceae were presumptively identified based on colony morphology, Gram stain and API^®^ 20C Aux (Biomerieux) for yeasts and API^®^ 20E (Biomerieux) for Enterobacteriaceae. *E. coli* was confirmed by PCR amplification of the *uspA* gene (Chen and Griffiths, 1998) (**Supplementary Figure S1D**). All isolates were stored at -80°C until further use.

### Antimicrobial Susceptibility Testing

The antimicrobial susceptibility tests included six antibiotics: amoxicillin/clavulanic Acid (AMC) 20/10 μg (BBL, Becton Dickinson, USA), sulphamethoxazole/trimethoprim (SXT) 25 μg (Oxoid Ltd., Basingstoke, Hampshire, England), cefotaxime (CTX) 30 μg (BBL, Becton Dickinson, USA), tetracycline (TET) 30 μg (BBL, Becton Dickinson, USA), gentamicin (GN) 10 μg (BBL, Becton Dickinson, USA), and neomycin (NE) 30 μg, (Oxoid Ltd., Basingstoke, Hampshire, England).

The susceptibility to antibiotics was tested against all bacterial isolates (*n* = 324) using the diffusion test proposed by Kirby–Bauer following the recommendations of the Clinical Laboratory Standards Institute guidelines (Clinical Laboratory Standards Institute [CLSI], 2012b) and the BSAC guide (Andrews and BSAC Working Party on Susceptibility Testing, 2008). In this analysis only the three *Candida* spp. were excluded. Organisms were incubated on Mueller Hinton (MH) agar plates at 35°C for 18–24 h in aerobic atmosphere. *E. coli* (ATCC-25922) and *S. aureus* (ATCC-25923) were used as quality control. The CLSI breakpoints were used for the interpretation of susceptibility to all microbial agents. The BSAC standard was applied to determine the breakpoint value of Neomycin (NE).

### Determination of Minimum Inhibitory Concentration of Copper (MIC-Cu)

The minimum inhibitory concentration of copper (MIC-Cu) was determined by the agar dilution method against 327 microorganisms isolated, applying standard bacteriological methods (Clinical Laboratory Standards Institute [CLSI], 2012a). Briefly, MH agar plates were supplemented with seven concentrations of copper (II) sulfate pentahydrate (CuSO_4_x5H_2_O) (Merck Millipore, Germany): 50, 125, 250, 375, 500, 750, and 1000 ppm of copper. Overnight culture of each microorganism was diluted to 1x10^6^ CFU/ml; 5 μL of this dilution was inoculated as spots with a microplate replicator. Each assay was performed in triplicate. The MIC-Cu was defined as the lowest concentration of copper at which no growth was observed following overnight incubation at 37°C.

### Inactivation of Microorganisms by Copper

The antibacterial effect of copper over time was evaluated in the four most prevalent strains [coagulase-negative *Staphylococci* (CNS) (MT-163), *E. coli* (Al-563), *S. aureus* (MT-359), and *S. uberis* (MT-360)] isolated from milk with clinical bovine mastitis. For this, an overnight culture for each bacteria was refreshed and grown until it reached an optical density of 1.0 (600 nm) in TSAYE broth (BBL, Becton Dickinson, USA) at 37°C. For the inactivation treatment, the same concentration of MIC-Cu for each microorganism (previously stated) and double the MIC-Cu were prepared in phosphate buffered saline PBS buffer (pH = 7.0) and inoculated with the above refreshed culture with concentration *ca*. 10^7^–10^8^ CFU/ml.

One-hundred micro liter of the suspension were taken at 0, 15, 30, 60, and 90 min after copper exposition and mixed with 900 μl of sterile PBS to stop the effect of the copper. One-hundred micro liter of this dilution as well as the same volume of serial dilutions were seeded in TSAYE agar and incubated at 37°C for 24 h for survivor counting. Experiments were performed in triplicate and sterile PBS was used as control medium.

### Statistical Analysis

To evaluate the significant relationships between species of microorganisms isolated and geographical region, we used the two sample proportion test (Stata V11.0). The level of significance used was *p* < 0.05.

The Dc value, defined as the time (min) required to decrease the microbial population by 1 log CFU/ml at a specific copper concentration, was calculated for all the assayed conditions mentioned in the previous section. For this purpose, the decimal logarithm of the number of survivors was plotted versus the time (min) to obtain the survival curves for each experimental condition. The curves were fitted using the freeware tool GInaFiT Add-In Microsoft Excel (Geeraerd et al., 2005). The *Dc* values were calculated from the inactivation rate (k_max_) from the best fit provided by GInaFiT (*Dc* = 2.303/kmax). A one way ANOVA was conducted to compare *Dc* values. ANOVA assumptions were evaluated using the Bartlett test (Zar, 1999) with StatGraphics Plus 5.0.

## Results

### Microorganisms Isolated from Bovine Mastitis

A total of 386 milk samples were collected, 274 from the southern region where 199 milk samples presented signs of subclininal mastitis and 75 of clinical mastitis. In the central region 112 samples were collected, 49 with subclinical mastitis and 63 samples with clinical mastitis.

**FIGURE 2:**
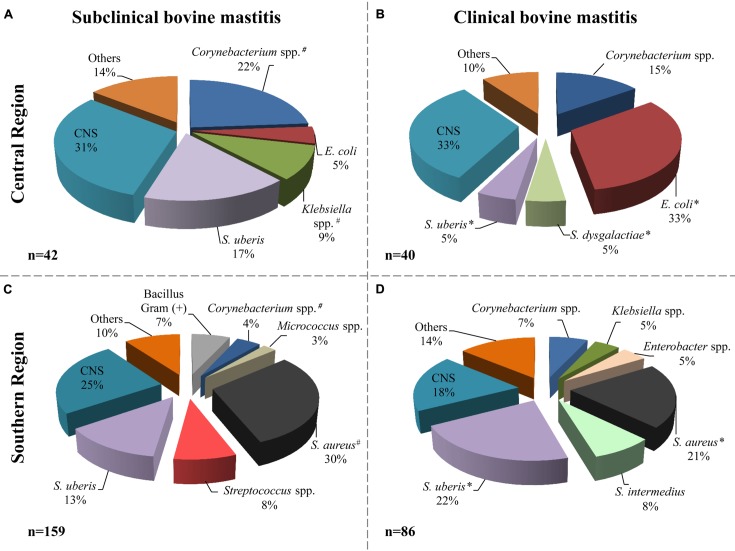
**Bacterial species distribution isolated from milk samples.** Microorganisms identified in subclinical (**A**, central; **C**, southern) and clinical (**B**, central; **D**, southern) mastitis milk samples. The central region includes 82 isolates and the southern region includes 245 isolates. The label “Others” includes microorganisms isolated with frequency ≤2% of the total. *^#^* indicate that the frequency of isolation of microorganisms, from subclinical mastitis, between the regions is significantly different (*p* ≤ 0.05). ^∗^ indicate that the frequency of isolation of microorganisms, from clinical mastitis, between the regions is significantly different (*p* ≤ 0.05). Others included: *Enterobacter cloacae*, *Enterococcus faecium*, *Streptococcus* spp., *Bacillus*, non-fermenting *Bacillus*, *Aeromona*, *Yersinia* sp., *Serratia* sp., and *Candida* spp.

According to the microbiological analysis of samples, the milk from cattle with mastitis was negative in 123 samples (32%); nine samples were excluded for contamination with three or more microorganisms. Of the remaining samples (*n* = 254) a total of 327 microorganisms were isolated (**Figure 2**); three of them were identified as *Candida* spp. from subclinical mastitis (one from the central region and two from the southern region). Different bacterial species were identified; the distribution related to the origin and the types of mastitis is shown in **Figure 2**.

In animals from the central region, CNS, *Corynebacterium* spp. and *S. uberis* were the most frequent agents detected in subclinical mastitis samples; 13 (31%), 10 (22%), and 7 (17%), respectively (**Figure 2A**). In the animals with clinical mastitis, *E. coli* and CNS (33%) were the agents most frequently identified (**Figure 2B**). For the samples from the southern region, *S. aureus*, CNS, and *S. uberis* were the most frequent bacteria, from both clinical and subclinical mastitis (**Figures 2C,D**). It can be observed in the same figure that *E. coli* was identified only in the central region with 4 and 33% in subclinical and clinical bovine mastitis, respectively. In addition, *S. aureus* had greater presence in the southern region, with 30 and 21% in subclinical and clinical subclinical bovine mastitis, respectively.

The frequency of *S. uberis* isolation was greater in the southern sample than in the central region (22 versus 5%, respectively) in milk samples from clinical bovine mastitis; however, in subclinical samples it was slightly greater in the central region with 17% than in the southern region with 13%. Other bacteria such as *Corynebacterium* spp. isolates were also identified in samples with frequencies of 22 and 15% in the central regions and 4 and 7% in the southern region from subclinical and clinical mastitis, respectively.

In order to find potential strategies to control bovine mastitis we evaluated the susceptibility of all microorganisms isolated to antibiotics and to copper.

#### Antibiotic Susceptibility

Antibiotic susceptibility was evaluated for 324 bacteria isolated (*Candida* spp. were excluded, since antifungal agents were not tested). The results of the susceptibility testing showed that 215 out of 324 (66%) isolates were sensitive to all antibiotics tested. A larger number of these susceptible isolates belonged to cows with subclinical mastitis (*n* = 140 isolates). The frequency of antimicrobial susceptibility to antibiotics is presented in **Supplementary Figure S2**. We conclude that in the central region, the antibiotics CTX, AMC, TET, and NE presented a resistance percentage greater than 10% in the isolates with clinical mastitis (intermediate and resistant). The AMC, NE, and GN antibiotics showed similar results in the isolates obtained in bovines with clinical mastitis from the southern region.

The results of antibiotic susceptibility for microorganisms with higher frequency of isolation in bovines with clinical mastitis showed that *E. coli* isolated from the central region had a variable frequency of resistance to the different antibiotics tested (**Table 1**). In particular, 7/13 (54%) isolates showed resistance to NE. *S. uberis* isolated from the southern region showed a high percentage of resistance to aminoglycosides GN and NE (47 and 58%). Some *S. uberis* were also resistant to CTX (26%). *S. aureus* and CNS isolates presented a low frequency of resistance to almost all antibiotics tested (**Table 1**).

**Table 1:** Percentage of antibiotic resistance for the most frequently identified isolates from clinical bovine mastitis milk samples.

	Central region			South region		
Resistance to	*Escherichia coli*		CNS	*Staphylococcus aureus*	Streptococcus *uberis*	CNS
	(*n*:13)		(*n*:13)	(*n*:18)	(*n*:19)	(*n*:16)
GN	15.4%		0	0	47.4%	0
NE	53.8%		15.4%	5.6%	57.9%	6.3%
CTX	15.4%		0	0	26.3%	6.3%
AMC	23.1%		0	0	0	0
TET	23.1%		7.7%	0	0	0
SXT	15.4%		0	0	0	0

#### Activity of Antimicrobial Copper

To assess the antimicrobial activity of copper, MIC-Cu was evaluated for all microorganisms isolated from milk with clinical and subclinical bovine mastitis. The results showed (**Supplementary Figure S3**) that a majority of isolates from clinical and subclinical samples were inhibited by 125 ppm or less copper (90/327) and 250 ppm of copper (122/327); however, there were two isolates that required 700 ppm [*Candida* spp. (subclinical) in the central region and *Streptococcus* spp. (clinical) in the southern region]. No growth was observed for any microorganism at 1000 ppm. Subclinical isolates showed slightly lower MIC-Cu values than those observed for clinical mastitis isolates in both regions sampled. The different behavior of bacteria species to MIC-Cu was apparently not related to origin (central or southern region) of milk samples (**Supplementary Figure S3**); in both cases nearly 93% of isolates were inhibited with less than 500 ppm of copper.

**FIGURE 3:**
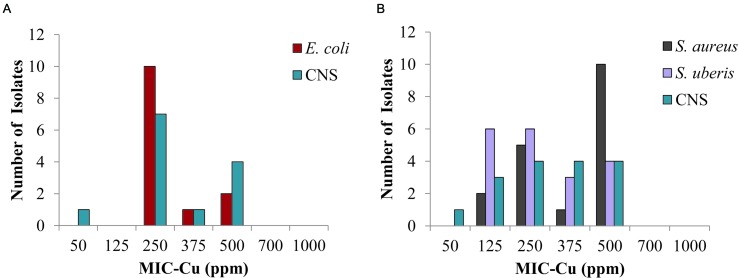
**Minimum inhibitory concentration of copper (MIC-Cu) value distribution for the most prevalent isolates from clinical bovine mastitis.** Isolates from: **(A)** central region and **(B)** southern region.

The results for the bacteria identified most frequently in clinical mastitis showed that 250 ppm of copper inhibited 77 and 62% of isolates of *E. coli* and CNS from the central region, respectively (**Figure 3A**), likewise, *S. aureus*, CNS, and *S. uberis* isolates (**Figure 3B**) from the southern region were inhibited 39, 50, and 63%, respectively (clinical mastitis). At 500 ppm copper all the isolates were completely inhibited (**Figure 3**).

**FIGURE 4:**
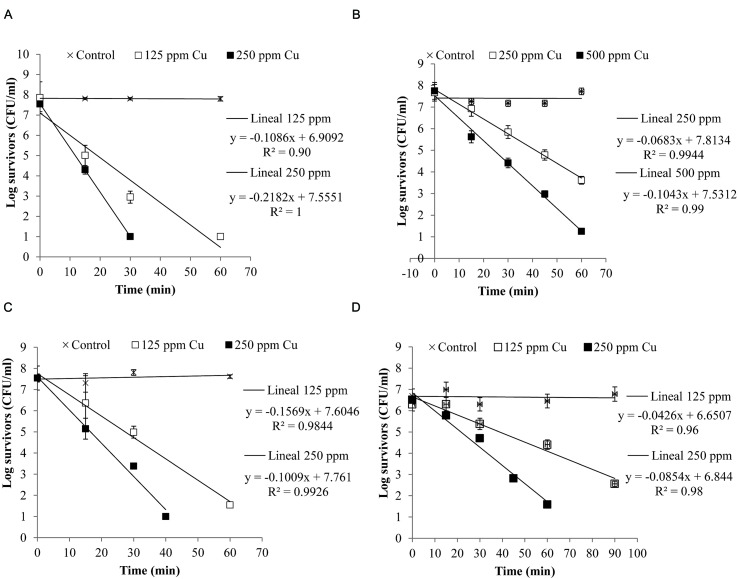
**Inactivation curves of copper of four isolates from clinical mastitis: (A) *Escherichia coli* (Al-563), (B) CNS (MT-163), (C) *S. aureus* (MT-359), and (D) *S. uberis* (MT-360).** The isolates were exposed to different concentrations of copper. Bacterial counts were analyzed by CFU assay. Each determination was made in triplicate.

**Figure 4** shows the inactivation curves of four strains belonging to the four most frequent pathogenic species which were isolated from clinical mastitis [*E. coli* (Al-563), CNS (MT-163), *S. aureus* (MT-359), and *S. uberis* (MT-360)]. It was observed that with less than 60 min of exposure to a copper concentration of 250 ppm *E. coli*, *S. aureus*, and *S. uberis* reduced their viability by almost 7 log CFU/ml, while CNS reduced its viability *ca*. 7 log CFU/ml after 60 min when it was exposed to 500 ppm of copper. The selected *E. coli* strain was the most susceptible to copper.

A log linear relationship was found between the logarithm of viable bacteria number and time of inactivation. A log-linear model (equations in **Figure 4**) of the experimental data resulted in high coefficients of determination (*R*^2^> 0.90).

The Dc values are shown in **Table 2**. It can be observed that for the same intensity of stress (250 ppm Cu), *E. coli* (*D*c = 4.6 min) was the least resistant, followed by *S. aureus* (*D*c = 6.37 min) and *S. uberis* (*D*c = 11.71 min). CNS showed the highest resistance value (*D*c = 14.64 min), meaning that it needs a longer time to reduce the population by 1 log.

**Table 2:** Dc values (min) for strains isolated from clinical bovine mastitis exposed to different copper concentrations.

	*D*c values (min)		
	Copper (ppm)		
	125	250	500
*E. coli*	9.21^a^ (0.03)	4.6^c^ (0.04)	–
CNS	–	14.64^d^ (0.012)	9.6^a^ (0.01)
*S. aureus*	9.91^a^ (0.01)	6.37^c^ (0.03)	–
*S. uberis*	23.47^b^ (0.01)	11.71^d^ (0.02)	–

*^a,b,c,d^The same letter indicates no significant difference (p > 0.05) under the same copper concentration. Values in brackets are standard error.*

The within-strain comparisons (**Table 2**) for different intensities of stress (ppm copper) showed that the *D*c values were significantly different in all cases (*p* < 0.05). Comparison between strains subjected to the same concentration of copper showed that the *D*c values of *E. coli* and *S. aureus* (125 and 250 ppm) were not significantly different (*p* > 0.05). The Dc value for 500 ppm copper in CNS was not significantly different from the *D*c value of 125 ppm for *E. coli* and *S. aureus*.

## Discussion

Bovine mastitis is a serious problem for dairy production worldwide. In this study we evaluated *in vitro* the antimicrobial effectiveness of copper solutions to inactivate microorganisms prevailing in bovine mastitis. The results showed that the prevailing agents are related to the geographical region. One study reported by Kalmus et al. (2011) indicated that *S. uberis and E. coli* are the main pathogens associated with clinical mastitis in Estonia. However, in Sweden *S. aureus* has high prevalence (21.3%) causing this pathology (Ericsson Unnerstad et al., 2009). In India, cows with subclinical mastitis showed a highly variable microbiota. Metagenomic analysis of the affected milk revealed that *E. coli* was the predominant microorganism in two cattle breeds studied, while *S. aureus* stood out in a third breed (Bhatt et al., 2012).

The present study explored the current frequency and diversity of pathogens involved in bovine mastitis in two geographical regions of Chile. The isolation frequency of *E. coli* was high in the central region, probably due to the permanent confinement system applied in this region. *S. aureus* was more frequently identified in the southern region where a free pasturage system prevails, as previously described (San Martin et al., 2002). These authors reported a similar prevalence of *S. uberis* in the two regions (near 3%). In contrast, in our study the frequency of detection of *S. uberis* was higher (22% clinical bovine mastitis) in the southern region under pasture feeding (**Figure 2**). These differences may imply that the etiology of bovine mastitis in Chile has undergone modification after 10 years. This change could be related to the type of feeding, the use of antibiotic therapy, environmental modifications or other factors as have been described in other countries (Zadoks and Fitzpatrick, 2009).

The use of antibiotics is frequent in the dairy industry to control bacteria-causing bovine mastitis (Oliver and Murinda, 2012; Royster and Wagner, 2015). Antibiotic-resistant bacteria can be favored as a result, becoming a serious problem for dairy farms. A number of studies have been undertaken to determine the frequency of antibiotic resistance in bovine mastitis (Tenhagen et al., 2006; Bengtsson et al., 2009; Oliver and Murinda, 2012; Saini et al., 2012b; Rato et al., 2013). In our study we also evaluated the antibiotic susceptibility of isolated microorganisms to antibiotics commonly used against bovine mastitis (**Supplementary Figure S2**). The results showed that on average 34% of the strains isolated were resistant to at least one antibiotic evaluated. In particular, *E. coli* isolates showed the highest resistance rate for almost all antibiotics tested. In addition, 69% of *E. coli* from the central region and clinical bovine mastitis strains were resistant to one or more antibiotics; similar results were recently reported (Saini et al., 2012a). In the current study many bacterial isolates showed resistance against neomycin (63/324; 19%); this antibiotic is commonly used as an intramammary antiseptic to treat bovine mastitis in Chile.

The use of iodine as teat dip is common in the national and international dairy industry; the main advantage being the low cost and the strong color that the product stains the nipple. However, the inactivation of iodine in the presence of organic matter and the limited efficacy are two negative characteristics of this product (Galton et al., 1984).

The use of copper as an alternative to prevent bovine mastitis appears as a novel and promising idea. Several reports confirm the antimicrobial potential of copper on various pathogens that cause nosocomial disease in humans (Warnes et al., 2010; Zhu et al., 2012). Our results show that a concentration as low as 250 ppm of copper inhibited bacterial growth in 65% of the isolates from bovine mastitis, including bacteria with a wide pattern of antibiotic resistance isolated from clinical mastitis (**Figure 3**). In addition, a higher copper concentration of 1000 ppm should ensure inactivation, meaning it prevents the multiplication of all the microorganisms involved in bovine mastitis (**Supplementary Figure S3**). Upton (1988) patented a method that proposed to control bovine mastitis by applying a solution of an organic compound containing a low copper concentration (50 ppm). His method was tested on only two bacterial species. In our study, we tested copper antimicrobial activity on eight different bacterial genera and yeasts (see **Supplementary Figure S3** and **Figure 3**).

In terms of the potential for cross-resistance between copper and clinical antibiotics, Cavaco et al. (2011) showed that the MIC of copper sulfate was not associated with methicillin resistance *S. aureus*. Similarly, we have not observed particularly high values of MIC-Cu for *E. coli* and *S. uberis* isolates (**Figure 3**). These species showed a higher percentage of strains resistant to antibiotics compared to the other species most frequently isolated from clinical bovine mastitis (**Table 1**). Yazdankhah et al. (2014) presented a review of different publications regarding susceptibility of copper and the profile of antibiotic susceptibility; the authors indicated that more evidence is necessary to clarify the link between copper tolerance and antibiotics resistance (Yazdankhah et al., 2014).

The four strains representing the species of microorganisms identified most frequently in clinical mastitis showed some variability in the response to the different concentrations of copper applied (**Figure 4**). Other studies have shown that there is a large variability in the number of survivors during inactivation (Aguirre et al., 2009; Aspridou and Koutsoumanis, 2015). This variability can be affected by the inoculum size, which also affects the inactivation time of the microorganism (Aspridou and Koutsoumanis, 2015).

We also demonstrated for the selected strains that the antibacterial effect of copper occurred in a short time (**Table 2** and **Figure 4**), which is important to consider for whether solutions containing this metal could be used as part of a mixture for dipping to control bovine mastitis. MICs chosen in this study allowed rapid inhibition of the strains of *E. coli*, *S. aureus*, and *S. uberis*, however, the strain chosen for CNS showed a slow decay. Koutsoumanis and Lianou (2013) showed that there was significant strain variability in response to stress. In our case it would be desirable to evaluate more strains to assess the biological variability of this response. However, in a practical scenario a concentration of 1000 ppm would inhibit 99% of the entire microbial load according to the results obtained with a Monte Carlo simulation (data not shown).

Previous studies on the antimicrobial properties of metallic copper surfaces have been reported; the effectiveness of different contents of copper alloys, temperatures, and other parameters have been also evaluated (Wilks et al., 2005; Michels et al., 2009). Also, other studies described the inactivation kinetics of a wide variety of microbes on copper surfaces (Mehtar et al., 2008; Espírito Santo et al., 2010; Weaver et al., 2010). Copper ions and oxidative stress play a role during contact of bacteria with copper surfaces (contact killing), inducing membrane damage in cells exposed to copper surfaces (Espírito Santo et al., 2008; Elguindi et al., 2009; Molteni et al., 2010).

## Conclusion

This study shows that copper inhibits bacterial multiplication of different species isolated from bovine mastitis, and it may be an attractive alternative to apply as a teat dip to control bovine mastitis in milk farms. A copper concentration of 1000 ppm inhibited almost all bacterial growth. However, further studies are needed to assess the microbial response to copper *in vivo*. The biological variability of individual bacterial cells and strains needs to be identified to optimize the copper concentration required to inhibit bacterial growth. The results of this study will be useful for developing a quantitative microbial risk assessment.

## Author Contributions

AR-J: Designed the research, bacterial identification, analysis of results, and write the paper. NC: bacterial identification, antimicrobial susceptibility, testing, and determination of MIC-Cu. JA: inactivation of microorganisms by copper and data analysis. MT: bacterial identification. GF: Designed the research and write the paper.

## Conflict of Interest Statement

The authors declare that the research was conducted in the absence of any commercial or financial relationships that could be construed as a potential conflict of interest.

## Footnotes

1 http://gochemless.com/pdf/EPA_Registers_Copper_Containing_Alloy_Products.pdf

## Supplementary material

FIGURE S1 **Polymerase chain reaction (PCR) amplification for confirmation of different bacterial species. (A)** *S. aureus*, **(B)** *S. uberis*, **(C)** *Corynebacterium* sp., and **(D)** *E. coli*.

FIGURE S2 **Frequency of antimicrobial susceptibility of bacteria isolated from the central region (A, *n* = 41; B, *n* = 40) and the southern region (C, *n* = 157; D, *n* = 86) of subclinical and clinical bovine mastitis, respectively**. White bars are susceptible, gray bars are intermediate and black bars resistant bacteria. Breakpoints (susceptible, intermediate, and resistant) were defined according to CLSI.

FIGURE S3 **Frequency of MIC-Cu values for microorganisms isolated from milk samples obtained from: (A) Central region (40 clinical and 42 subclinical mastitis) and (B) Southern region (86 clinical and 159 subclinical mastitis)**. Gray bars represent the frequency of microorganism isolated from subclinical mastitis and black bars are from clinical mastitis. Each determination was performed in triplicate.
