# Supplementary figures and images for: Antibacterial Effect of Copper on Microorganisms Isolated from Bovine Mastitis
Source: Front Microbiol. 2016 Apr 28;7:626. doi: 10.3389/fmicb.2016.00626 (PMC4848319; doi:10.3389/fmicb.2016.00626)

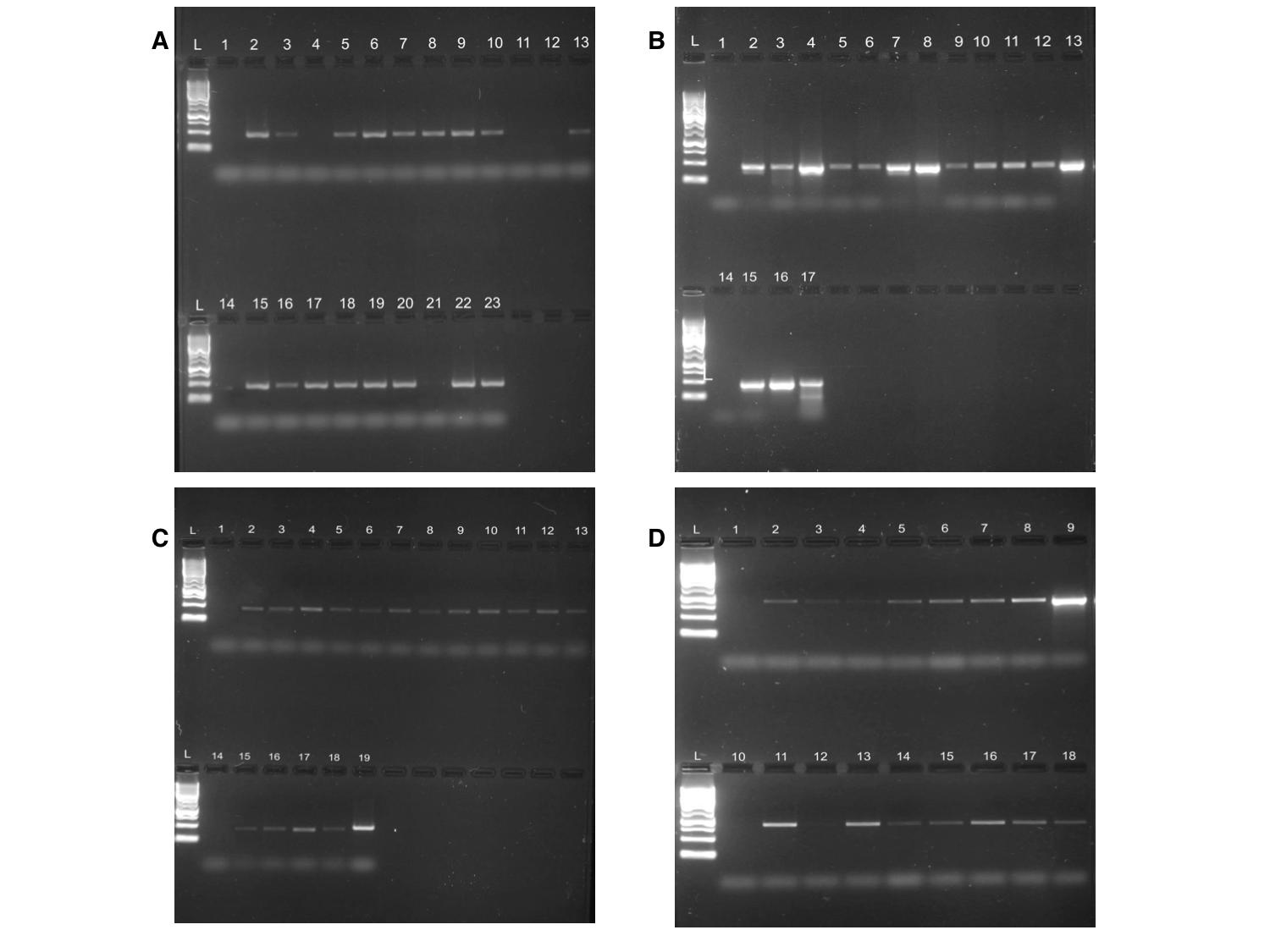

Supplement: FIGURE S1 — Polymerase chain reaction (PCR) amplification for confirmation of different bacterial species. (A) S. aureus, (B) S. uberis, (C) Corynebacterium sp., and (D) E. coli. [file Image_1.TIFF]

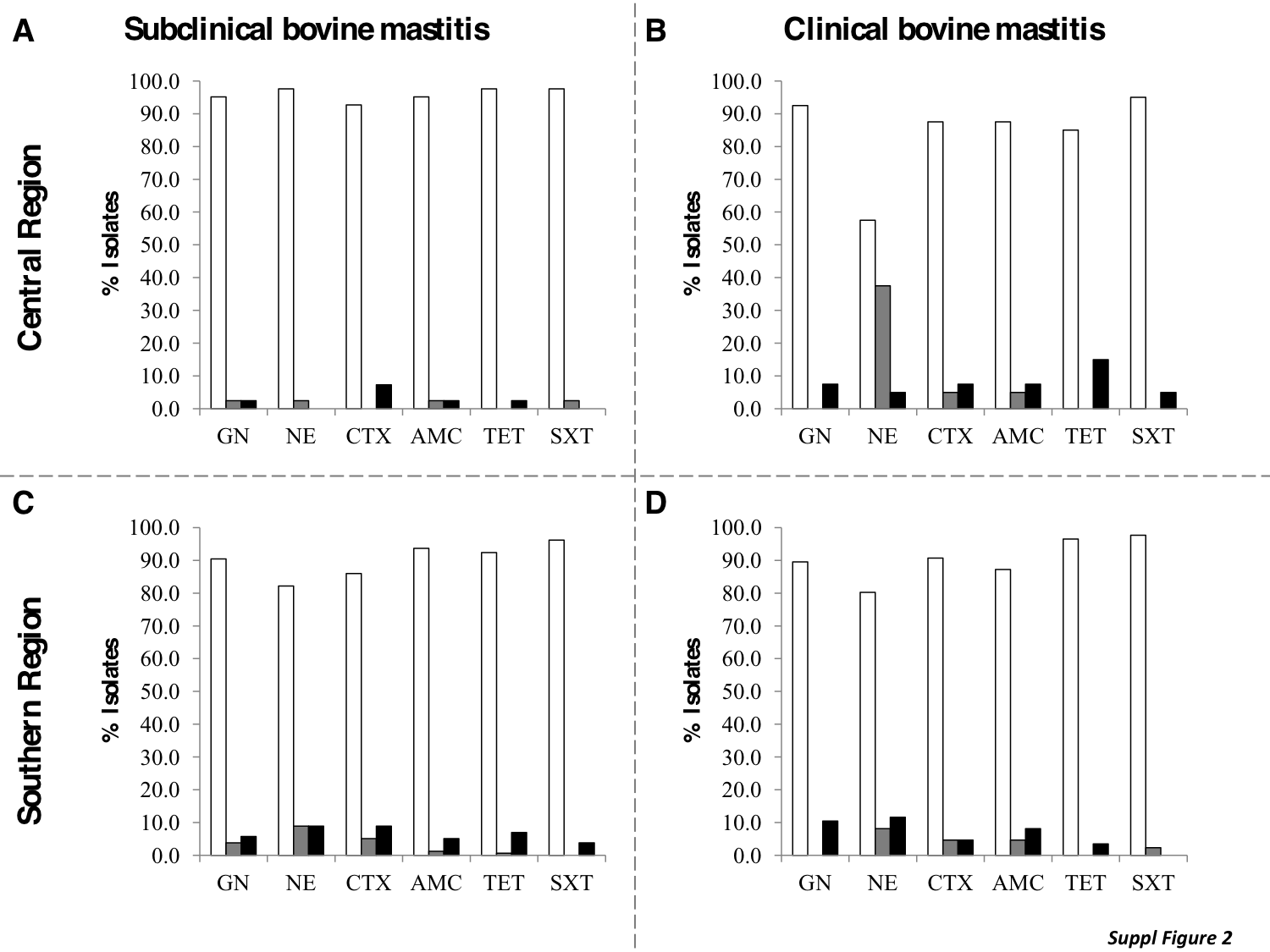

Supplement: FIGURE S2 — Frequency of antimicrobial susceptibility of bacteria isolated from the central region (A, n = 41; B, n = 40) and the southern region (C, n = 157; D, n = 86) of subclinical and clinical bovine mastitis, respectively. White bars are susceptible, gray bars are intermediate and black bars resistant bacteria. Breakpoints (susceptible, intermediate, and resistant) were defined according to CLSI. [file Image_2.TIFF]
